# Dorsal and ventral striatal dopamine D1 and D2 receptors differentially modulate distinct phases of serial visual reversal learning

**DOI:** 10.1038/s41386-020-0612-4

**Published:** 2020-01-15

**Authors:** Júlia Sala-Bayo, Leanne Fiddian, Simon R. O. Nilsson, Mona E. Hervig, Colin McKenzie, Alexis Mareschi, Maria Boulos, Peter Zhukovsky, Janet Nicholson, Jeffrey W. Dalley, Johan Alsiö, Trevor W. Robbins

**Affiliations:** 1 Department of Psychology and Behavioural and Clinical Neuroscience Institute, University of Cambridge, Cambridge, CB2 2EB UK; 2 Boehringer Ingelheim Pharma GmbH & Co. KG, Div. Research Germany, Biberach an der Riß, Germany; 3 Department of Psychiatry, University of Cambridge, Cambridge, CB2 2QQ UK

**Keywords:** Cognitive neuroscience, Psychology, Psychiatric disorders, Learning and memory

## Abstract

Impaired cognitive flexibility in visual reversal-learning tasks has been observed in a wide range of neurological and neuropsychiatric disorders. Although both human and animal studies have implicated striatal D_2_-like and D_1_-like receptors (D2R; D1R) in this form of flexibility, less is known about the contribution they make within distinct sub-regions of the striatum and the different phases of visual reversal learning. The present study investigated the involvement of D2R and D1R during the early (perseverative) phase of reversal learning as well as in the intermediate and late stages (new learning) after microinfusions of D2R and D1R antagonists into the nucleus accumbens core and shell (NAcC; NAcS), the anterior and posterior dorsomedial striatum (DMS) and the dorsolateral striatum (DLS) on a touchscreen visual serial reversal-learning task. Reversal learning was improved after dopamine receptor blockade in the nucleus accumbens; the D1R antagonist, SCH23390, in the NAcS and the D2R antagonist, raclopride, in the NAcC selectively reduced early, perseverative errors. In contrast, reversal learning was impaired by D2R antagonism, but not D1R antagonism, in the dorsal striatum: raclopride increased errors in the intermediate phase after DMS infusions, and increased errors across phases after DLS infusions. These findings indicate that D1R and D2R modulate different stages of reversal learning through effects localised to different sub-regions of the striatum. Thus, deficits in behavioral flexibility observed in disorders linked to dopamine perturbations may be attributable to specific D1R and D2R dysfunction in distinct striatal sub-regions.

## Introduction

Cognitive flexibility, the ability to adapt behavior to changes in the environment, is impaired in a wide range of neurological and neuropsychiatric disorders, including schizophrenia [1], obsessive-compulsive disorder (OCD) [2], Parkinson’s disease (PD) [3] and substance use disorder [4]. Such cognitive dysfunction can be evaluated in reversal-learning tasks. Converging evidence from such tests implicates dopamine (DA) as an important modulator of reversal learning. For instance, systemic blockade or agonism of D_2_-like receptors (D2R) impairs reversal learning in vervet monkeys and rats [5, 6], while D2R knockout mice show deficiencies in initial visual discrimination and in reversal learning [7]. In contrast, pharmacological activation of D_1_-like receptors (D1R) impaired early phases of reversal learning [8], whereas D1R antagonism did not alter reversal learning performance [5]. In healthy humans, repeat variations in the dopamine transporter gene, *DAT1*, have been linked to performance during the early, perseverative phase of reversal learning, when prior beliefs about the stimulus-reward outcomes still guide behavior, whereas accuracy during later phases, when new learning takes place, showed no such link [9].

The main sub-regions of the dorsal striatum, namely the caudate nucleus and the putamen in primates and the dorsomedial and dorsolateral striatum in rodents (DMS; DLS), have also been differentially linked to reversal learning. Recent evidence suggests that pharmacological inactivation of the putamen and caudate nucleus differentially affect serial visual reversal learning in marmoset monkeys [10]. Furthermore, D2R availability in these sub-regions of vervet monkeys is associated with reversal learning performance [11]. Importantly, the DMS appears strongly linked to the early, perseverative phase of reversal, whereas the DLS becomes engaged during later stages [12]. This is perhaps in line with the view that the DLS mediates stimulus-response habits whereas the DMS—especially the anterior over the posterior DMS (aDMS; pDMS; [13], but see [14])—is more strongly associated with goal-directed actions [15]. Both forms of control over instrumental behavior are likely necessary for implementing a new strategy following contingency reversal, specifically the ability to suppress prepotent, perhaps habitual, responding to the previously rewarded (and now unrewarded) stimulus, and flexibly learn to select, via goal-directed behavior, the previously unrewarded (now rewarded) option [16].

In the ventral striatum, previous studies have shown that increased dopaminergic tone in the nucleus accumbens (NAc), or infusions of a D2R agonist (quinpirole) into this area impaired reversal learning in rats [17], whereas infusions of a D1R agonist (SKF81297) disrupted set-shifting by increasing perseverative behavior [17, 18]. Lesions of the NAc disrupted initial stimulus discrimination and reversal learning [19, 20], including spatial, but not visual, reversal learning in monkeys [21], and pharmacological inactivation impaired probabilistic learning in rats [22]. However, other studies report no effect of NAc interventions on such flexibility [23, 24]. This discrepancy may be explained by the heterogeneity of the NAc with the core and shell sub-regions (NAcC; NAcS) contributing differentially to attention [25, 26] and impulsivity-related behaviors [27–29], with these NAc sub-regions often being suggested to play opposite roles in modulating behavior. For instance, inactivation of the NAcS impaired probabilistic reversal performance in rats, identifying a key role for this nucleus in using probabilistic reward feedback to facilitate discriminative learning and flexibility, whereas inactivation of the NAcC, while not affecting performance accuracy did cause a general slowing of approach toward the response levers [22].

Taken together, this evidence suggests a general pattern of impaired reversal learning when DA activity is low in the dorsal striatum and when the dopaminergic tone is elevated in the ventral striatum. However, there is no clear evidence of the role of D1R and D2R in different sub-regions of the striatum in visual reversal learning or of their involvement in its different learning phases.

We therefore sought to investigate whether D1R and D2R differentially affect reversal learning both across different striatal sub-regions, including DLS, aDMS, pDMS, NAcC and NAcS, and on the different phases of reversal learning by exploring the behavioral effects of local administration of a D2R antagonist and a D1R antagonist using a recently established touchscreen task for rats [30].

## Materials and methods

### Subjects

The subjects were 82 male Lister-Hooded rats (Charles River, UK) initially housed in groups of up to 4 under humidity- and temperature-controlled conditions and a 12:12-h light-dark cycle (lights off at 0700 h). Following implantation of guide cannulae, animals were singly housed. Rats were ≈300 g at the beginning of training and were maintained at >85% of their free-feeding weight by food restriction (19 g/day of Purina chow). Water was provided ad libitum. The number of animals used for each experiment is shown in Table 1. The work was carried out under a UK Home Office Project license (PPL 70/7548) in accordance with the UK Animals (Scientific Procedures) Act 1986 and local ethical review at Cambridge University.

**Table 1:** Coordinates and group size for the different striatal sub-regions and DA receptor antagonists, raclopride (D2R) and SCH23390 (D1R).

Coordinates	NAcC	NAcS	aDMS	pDMS	aDLS
*Guide cannulas*					
AP	+1.2	+1.6	+1.2	−0.4	+1.2
ML	±1.9	±0.75	±1.9	±2.6	±3.5
DV	−1.9	−1.9	−1.9	−2.4	−2.4
*Injectors*					
DV	−6.9	−6.9	−4.4	−4.4	−4.4
*n*					
Raclopride	22	10	15	15	11
SCH23390	13	9	15	10	5

AP and ML were measured from bregma and DV from dura

### Experimental procedures

Surgeries and microinfusion procedures are described in the Supplementary Materials and Methods.

### Behavioral pre-training

**Fig. 1:**
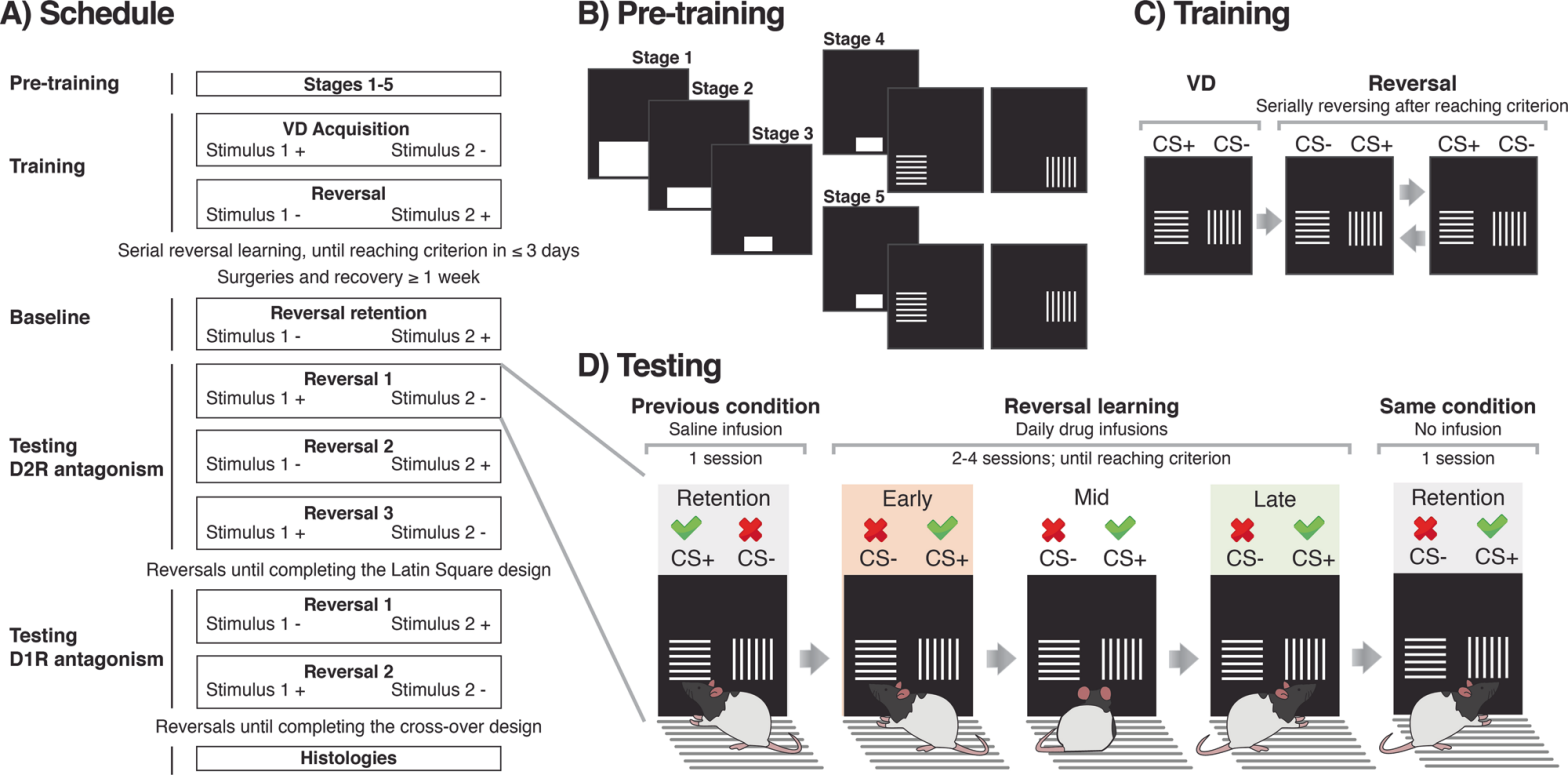
Schematic representation of the task. **a** Behavioral training and testing protocol. The rewarded stimulus is represented as a + and the unrewarded stimulus as a −. Stimuli were vertical or horizontal bars and were counterbalanced as CS+ or CS− across rats. **b** Diagram of pre-training stages, from 1 to 5. Stimulus presentation in stages 4 and 5 was preceded by the same starting box from stage 3. Only one of the two stimuli appeared at any one time. Position (i.e. left/right) was pseudo-randomized. **c** Representation of the stimuli during visual discrimination (VD) and reversal learning. Criterion was reached at a performance of ≥24/30 correct responses, which represents a performance at or above 80%. After criterion was met during both reversal learning and in two retention sessions, conditions changed again. **d** Flowchart of the testing procedure and phases of reversal learning. Phases depended on performance within sessions. After reversal, during the early phase performance was lower than 11 correct trials out of a set of 30 trials, as animals tended to perseverate on the previously CS+, now CS−. After some trials, performance improved, and animals reached the so-called mid, intermediate or random phase, before reaching the late or learning phase, in which they have learnt to approach the new CS+ (>19/30 correct responses) [30, 31].

All software was written by Dr. A. C. Mar [30]. Rats were initially trained to touch the screens with daily sessions of 60 min or 100 trials. Pre-training consisted of five stages with gradually increased difficulty (Fig. 1b). Briefly, in stage 1, a large white horizontal square ‘start-box’ (15 × 9 cm) was presented in the bottom center of the screen, and touching it was associated with reward (45 mg sucrose pellet; TestDiet 5UTL; Sandown Scientific, Middlesex, UK). The size of the ‘start box’ decreased throughout the stages until measuring 3 × 4 cm in stage 3. Animals were moved to the next stage when reaching 100 responses/rewards per session. In stage 4, touching the white box was not reinforced but led to the presentation of a visual stimulus (vertical or horizontal bars) with a pseudo-random spatial placement, left or right. The same stimulus was not displayed on the same side for more than three consecutive trials to avoid side-biasing. Responding to the stimulus was reinforced, whereas the blank side led to the illumination of the house-light for a 5 s time-out (TO) period. After collecting the reward, there was an inter-trial interval (ITI) of 5 s. In stage 5, the stimuli were presented slightly higher to avoid accidental touches e.g. with the tail. The criterion to move from stages 4 and 5 was reaching ≥80% of correct responses per session.

### Visual discrimination training

After the initial training stages, subjects were trained on a visual two-choice discrimination task (Fig. 1). Touching the square ‘start-box’ triggered the simultaneous presentation of two stimuli (vertical and horizontal bars), determined pseudo-randomly on either left or right side of the screen [30]. The start-box procedure was used to ensure the central position of the animal before the choice phase. Responses to one stimulus (CS+) were associated with reward and collecting the reward initiated the next ITI. In contrast, responses to the other stimulus (CS−) were not rewarded and led to a house light-signaled TO. The response window after stimulus presentation was set to 10 s. After this time, the trial was considered as an omission and led to a new ITI. The session ended after 250 trials, 150 rewards or 1 h, whichever came first. Criterion for discrimination learning was set to 24 correct responses out of 30 consecutive trials. Once acquired within any session, rats were given a retention session with the same reward contingencies to ensure they had reliably acquired the visual discrimination.

### Serial visual reversal learning

Following acquisition of visual discrimination, animals were trained in serial visual reversal learning (Fig. 1c). After the discrimination and retention sessions, contingencies reversed so the previous CS+ was then CS− and vice versa. Rats were required to respond to the new CS+ until reaching the discrimination criterion (≥24/30 correct responses). After reaching criterion, an extra retention session was run. Additional reversals were performed until the rats were able to attain the criterion within 3 daily sessions. When this was met, rats underwent surgery prior to testing. A retention session was run before each reversal and after reaching the criterion (Fig. 1d), both in training and testing.

### Data analysis

The main dependent variables were the number of errors and trials to criterion (≥24/30 correct responses). Omissions, latencies to respond and latencies to collect the reward were additionally analyzed. Data from each reversal were collapsed over days. Trial outcomes were classified in three different phases: early, mid or late, depending on the performance over a running window of 30 consecutive trials [30, 31]. If animals had a significant bias (binomial distribution probabilities) towards the previously positive stimulus (<11/30 correct responses), performance was considered to belong to the early phase, in which animals exhibited mainly perseverative responses. If their performance instead showed a significant preference for the currently rewarded stimulus (>19/30 correct responses) it was considered as the late phase, in which animals moved closer to criterion for learning the reversed contingency. Performance in-between these thresholds was classified as intermediate or mid-phase, prior to acquisition of the new learned association. Data from all trials after the rats had reached the final learning criterion (≥24/30 correct responses) were excluded from the analysis.

Statistical tests were performed with RStudio, version 1.2.1335 (RStudio, Inc). Errors were square-root transformed and latencies log transformed to ensure normality. Data were then subjected to Linear Mixed-Effects Model analysis with the lmer package in R. The model contained three fixed factors (dose, phase, region) and one factor (subject) modeled as a random slope to account for individual differences between rats across phases (i.e. individual learning curves). Significance was considered at *α* = 0.05. The normality of residuals was confirmed with a quantile-quantile plot (QQ plot) and model fitting was tested with a Chi-squared test. When significant three-way interactions were found, further analysis was performed by conducting separate multilevel models on “dose” and “phase” for each region. In the absence of significant three-way interactions, two-way Dose × Region interactions were explored further. Analysis was followed by post hoc Tukey’s corrected pairwise comparisons.

## Results

### Histology

The ventral-most locations of injectors are included in each of the data figures. Rats were excluded from the study if the injector cannulas were positioned outside the target areas (*n* = 3 pDMS, *n* = 5 DLS and *n* = 1 NAcC). Final group sizes with verified injector positions for each of the drug groups and targeted coordinates are shown in Table 1.

### Effects of intra-striatal infusions of the D2R antagonist raclopride and the D1R antagonist SCH23390

Across all behavioral variables we found no significant differences between the aDMS and pDMS. We therefore combined these two regions as ‘DMS’ for subsequent analysis. Separate data for each of these regions are given in the Supplementary Material online.

**Fig. 2:**
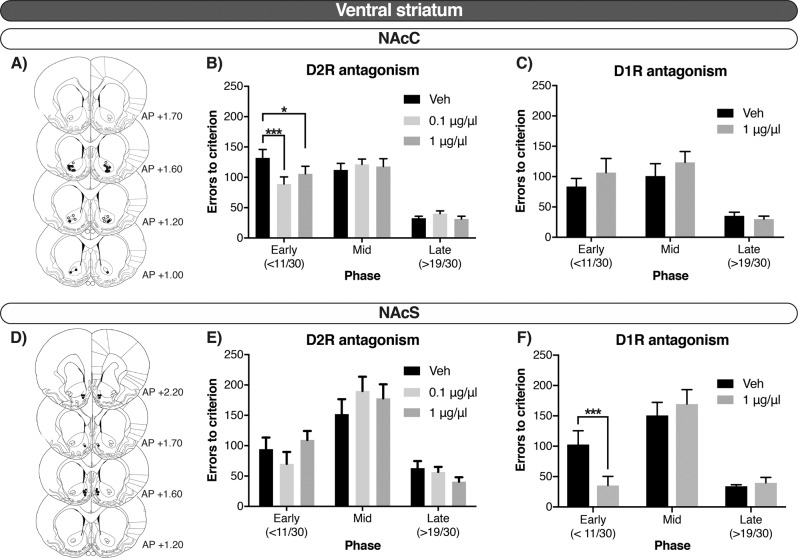
In the ventral striatum, reversal learning was modulated via D1R in the NAcC and D2R in the NAcS during early stages of reversal learning. **a**, **d** Injector tip placements. Closed circles represent rats that received both raclopride and SCH23390; open circles represent rats that received only raclopride. **b**, **e** Errors to criterion by phase—early, mid and late—after the D2R antagonist, raclopride, in the NAcC and NAcS, respectively. **c**, **f** Errors to criterion by phase—early, mid and late—after the D1R antagonist, SCH23390, in the NAcC and NAcS, respectively. Errors until reaching criterion of a high performance (>24/30 correct responses) are collapsed over reversals. Data shown as mean ± SEM. *p < 0.05. ***p < 0.001.

**Fig. 3:**
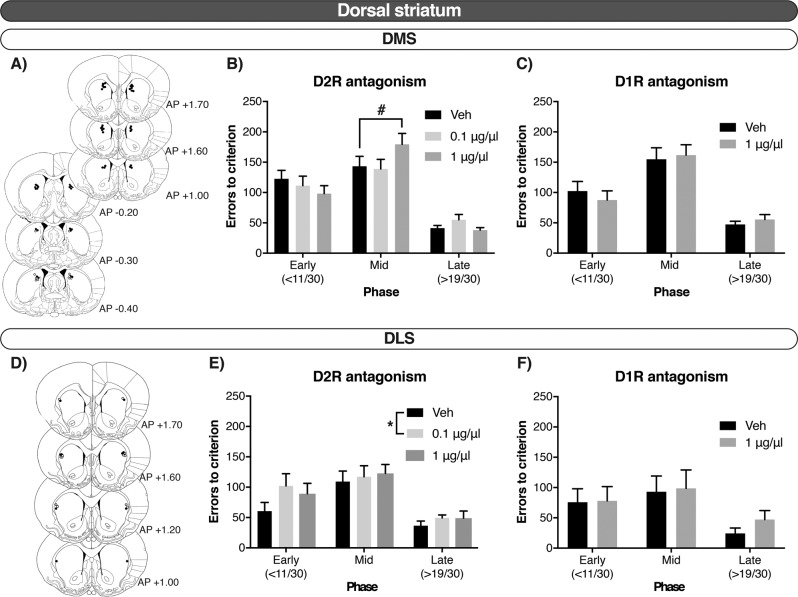
In the dorsal striatum, reversal learning was modulated via D2R in the DMS during the intermediate phase, and in the DLS during across all the phases of reversal learning. **a**, **d** Injector tip placements. Closed circles represent rats that received both raclopride and SCH23390; open circles represent rats that received only raclopride. **b**, **e** Errors to criterion by phase—early, mid and late—after the D2R antagonist, raclopride, in the DMS and DLS, respectively. **c**, **f** errors to criterion by phase—early, mid and late—after the D1R antagonist, SCH23390, in the DMS and DLS, respectively. Errors until reaching criterion of a high performance (>24/30 correct responses) are collapsed over reversals. Data shown as mean ± SEM. #*p* = 0.05. *p < 0.05.

Figures 2 and 3 indicate that whereas local infusions of the D2R antagonist raclopride improved early stages of reversal learning when administered into the NAcC, they impaired reversal when given in the dorsal striatum, both in the DMS (mid-phase) and DLS (across phases). In contrast, D1R antagonism in the NAcS improved the early phase of reversal learning but did not affect the number of errors when administered into the NAcC.

Analysis for both raclopride and SCH23390 treatments substantiated that the effect of drugs varied across regions and phases of the reversal task. For the number of errors committed we found a significant Dose × Phase × Region interaction after both raclopride (*F*_12, 479.990_ = 4.109, *p* = 0.005) and SCH23390 (*F*_6, 191.999_ = 4.109, *p* < 0.001) treatment. This was matched by significant Dose × Phase × Region interactions in number of trials per phase after antagonists administration (Raclopride: *F*_12, 407.990_ = 5.300, *p* < 0.001; SCH23390 *F*_6, 192.010_ = 3.280, *p* = 0.004). In addition, there was a significant Dose × Phase × Region interaction on omissions after SCH23390 microinfusions (*F*_6, 232.089_ = 11.512, *p* < 0.001), whereas no such effect was detected for raclopride (ns). On latencies, we observed no three-way interactions, but a number of Dose × Region interactions. Thus, we found a significant Dose × Region interaction in latencies to collect after infusions of Raclopride (*F*_6, 469.120_ = 3.511, *p* = 0.002), and both in latencies to collect and to respond with administration of SCH23390 (*F*_3, 221.033_ = 19.275, *p* < 0.001; *F*_3, 220.847_ = 24.379, *p* < 0.001, respectively).

#### Effects of D1R and D2R antagonism in the ventral striatum

Since the three-way interactions were significant, separate multilevel models were used to ascertain the phase-dependency of the drug effects in each region separately. Thus, in the NAcC there was a Dose × Phase interaction on the number of errors after raclopride infusions (*F*_4, 126.01_ = 3.905, *p* = 0.005). Post hoc analysis revealed that raclopride selectively improved performance during the early phase of reversal learning when infused in the NAcC at 0.1 μg/μl and 1 μg/μl, compared to vehicle control (*p* < 0.001 and *p* = 0.028, respectively; Fig. 2b). In the NAcS, there was also a Dose × Phase interaction for errors (*F*_4, 63.005  _= 3.813, *p* = 0.008), but pairwise comparisons revealed that no dose differed from the vehicle-control group (ns). There was thus no clear effect of raclopride when infused into the NAcS (Fig. 2e).

In contrast, analysis on the number of errors committed after SCH23390 infusions identified a significant Dose × Phase interaction after NAcS infusions (*F*_2, 31.997_ = 25.616, *p* < 0.001). Post hoc analyses showed that D1R antagonism into the NAcS selectively decreased perseveration in the early phase compared with the vehicle condition (*p* < 0.001; Fig. 2f). No main effect of Dose or a Dose × Phase interaction was observed after SCH23390 infusions into the NAcC (ns; Fig. 2c).

The above results on the number of errors committed after infusions into the NAcC and NAcS were similar when trials were analyzed instead. Specifically, the interactions Dose × Phase were significant for raclopride in the NAcC (*F*_4, 126_ = 3.402, *p* = 0.011); and for SCH23390 in the NAcS (*F*_2, 32_ = 20.328, *p* < 0.001) but not the NAcC (ns).

Table 2B shows that in the NAcC, SCH23390 strongly affected the number of omissions (Dose × Phase: *F*_2, 58.492_ = 11.838, *p* < 0.001). Post hoc analysis showed that SCH23390 selectively increased the number of omissions in the early phase (*p* < 0.001), with no significant effect during the mid or late phases (ns). No such effect was detected after NAcS infusions of SCH23390, or after raclopride infusions into either the NAcC or the NAcS (Table 2). SCH23390 infusions also prolonged the latencies to collect the reward and to respond to the stimuli in both sub-regions regardless of the phase (Dose: in Collect, NAcC: *F*_1, 57.096_ = 85.205, *p* < 0.001, and NAcS: *F*_1, 31.062_ = 99.382, *p* < 0.001; in Respond, NAcC: *F*_1, 57.181_ = 64.593, *p* < 0.001, and NAcS: F_1, 31.082_ = 7.838, *p* = 0.009). Raclopride had no effect on these variables in either NAcC or NAcS (Table 2A).

**Table 2:** D1R antagonism increased omissions when infused in the NAcC.

Region	Dose	Omissions			Latency to collect			Latency to respond		
		Early	Mid	Late	Early	Mid	Late	Early	Mid	Late
**A) Raclopride**										
*DMS*	Veh	1.43 ± 0.43	1.33 ± 0.30	0.23 ± 0.12	3.06 ± 0.03	3.04 ± 0.05	2.91 ± 0.03	3.00 ± 0.02	3.00 ± 0.02	3.01 ± 0.02
	0.1	1.00 ± 0.39	1.20 ± 0.37	0.43 ± 0.18	*3.12* ± *0.05*^a^	*3.08* ± *0.04*^a^	*2.95* ± *0.03*^a^	3.00 ± 0.02	3.00 ± 0.02	3.00 ± 0.02
	1	2.63 ± 0.83	2.83 ± 0.74	0.63 ± 0.22	*3.23* ± *0.05*^b^	*3.10* ± *0.03*^b^	*3.01* ± *0.04*^b^	3.07 ± 0.02	3.04 ± 0.02	3.06 ± 0.02
*DLS*	Veh	0.60 ± 0.40	0.30 ± 0.21	0.50 ± 0.50	3.01 ± 0.04	2.93 ± 0.04	3.01 ± 0.09	3.02 ± 0.07	2.98 ± 0.02	2.99 ± 0.04
	0.1	0.50 ± 0.50	0.40 ± 0.13	0.10 ± 0 13	3.01 ± 0.06	2.96 ± 0.06	2.89 ± 0.07	2.99 ± 0.02	2.98 ± 0.02	2.96 ± 0.02
	1	0.90 ± 0.50	0.20 ± 0.13	0.20 ± 0.13	3.07 ± 0.06	3.03 ± 0.06	2.92 ± 0.07	3.04 ± 0.02	3.01 ± 0.02	2.99 ± 0.02
*NAcC*	Veh	2.91 ± 0.88	1.14 ± 0.33	0.36 ± 0.14	3.22 ± 0.05	3.09 ± 0.03	3.08 ± 0.04	3.08 ± 0.02	3.06 ± 0.02	3.07 ± 0.02
	0.1	2.05 ± 0.64	1.41 ± 0.40	0.86 ± 0.27	3.19 ± 0.06	3.09 ± 0.03	3.03 ± 0.04	3.10 ± 0.03	3.08 ± 0.02	3.09 ± 0.02
	1	3.68 ± 1.09	2.59 ± 0.89	0.36 ± 0.14	3.24 ± 0.04	3.15 ± 0.04	3.08 ± 0.50	3.13 ± 0.03	3.10 ± 0.03	3.08 ± 0.02
*NAcS*	Veh	1.00 ± 0.70	0.50 ± 027	0.10 ± 0.10	3.26 ± 0.05	3.17 ± 0.04	3.08 ± 0.05	2.98 ± 0.03	2.91 ± 0.03	2.90 ± 0.03
	0.1	0.30 ± 0.15	0.20 ± 0.22	0.10 ± 0.10	3.21 ± 0.05	3.15 ± 0.04	3.03 ± 0.05	2.90 ± 0.05	2.90 ± 0.05	2.91 ± 0.04
	1	0.30 ± 0.15	0.50 ± 0.22	0.10 ± 0.10	3.17 ± 0.05	3.13 ± 0.04	3.04 ± 0.05	2.97 ± 0.05	2.91 ± 0.05	2.91 ± 0.04
**B) SCH23390**										
*DMS*	Veh	1.20 ± 0.54	1.56 ± 0.53	0.24 ± 0.09	3.15 ± 0.03	3.08 ± 0.03	2.99 ± 0.03	3.01 ± 0.02	3.00 ± 0.02	3.01 ± 0.02
	1	1.36 ± 0.55	2.00 ± 0.75	0.60 ± 0.33	*3.27* ± *0.05*^b^	*3.23* ± *0.05*^b^	*3.15* ± *0.04*^b^	3.02 ± 0.02	3.02 ± 0.02	3.04 ± 0.02
*DLS*	Veh	0.00 ± 0.00	0.20 ± 0.20	0.00 ± 0.00	3.07 ± 0.08	3.03 ± 0.07	3.15 ± 0.09	2.99 ± 0.04	3.00 ± 0.05	2.99 ± 0.04
	1	1.40 ± 1.16	0.17 ± 0.17	0.00 ± 0.00	3.19 ± 0.06	3.16 ± 0.06	3.17 ± 0.11	3.00 ± 0.04	3.00 ± 0.03	3.02 ± 0.05
*NAcC*	Veh	0.64 ± 0.24	1.54 ± 0.71	0.69 ± 0.47	3.23 ± 0.09	3.12 ± 0.07	3.06 ± 0.06	3.04 ± 0.03	3.01 ± 0.03	3.05 ± 0.03
	1	*30.08* ± *10.22*^c^	8.08 ± 2.63	1.92 ± 0.74	*3.79* ± *0.07*^b^	*3.57* ± *0.07*^b^	*3.54* ± *0.10*^b^	*3.23* ± *0.04*^b^	*3.22* ± *0.05*^b^	*3.25* ± *0.03*^b^
*NAcS*	Veh	0.13 ± 0.13	0.50 ± 0.38	0.00 ± 0.00	3.09 ± 0.04	3.05 ± 0.04	3.00 ± 0.05	2.99 ± 0.04	2.97 ± 0.04	2.97 ± 0.05
	1	0.00 ± 0.00	1.00 ± 0.42	0.88 ± 0.30	*3.47* ± *0.07*^b^	*3.34* ± *0.06*^b^	*3.32* ± *0.06*^b^	*3.01* ± *0.04*^b^	*3.01* ± *0.04*^b^	*3.07* ± *0.04*^b^

Effects of microinfusions of the A) D2R antagonist, raclopride (0, 0.1, 1 µg/µl) and B) D1R antagonist, SCH23390 (0, 1 µg/µl), in the DMS, DLS, NAcC and NAcS during the different phases of visual reversal learning (early, mid and late) as omissions, latencies to collect the reward and latencies to respond. Data are mean ± SEM. Latencies are presented as log-transformed values

^a^*p* < 0.01 vs vehicle treatment, Tukey post hoc after significant Dose × Region interaction

^b^*p* < 0.001 vs vehicle treatment, Tukey post hoc after significant Dose × Region interaction

^c^*p* < 0.001 after significant Dose × Phase × Region interaction

#### Effects of D1R and D2R antagonism in the dorsal striatum

The potential effects of drug infusions into the dorsal striatum were analysed next. There was a phase-dependent effect of raclopride in the DMS (Dose × Phase: *F*_4, 196.002_ = 3.574, *p* = 0.008). As can be seen in Fig. 3b, post hoc analysis showed that, in this region, the high dose (1.0 µg/µl) of raclopride marginally induced a significant impairment in the mid phase (*p* = 0.050) versus saline. There was no significant Dose × Phase interaction after raclopride infusions into the DLS (ns), although a main effect of Dose and Phase was observed (Phase: *F*_2, 12.057_ = 17.472, *p* < 0.001; Dose: *F*_2, 70.008_ = 3.764, *p* = 0.028). We explored this further and identified the main effect was driven by the low dose of raclopride across all the phases of reversal learning (Fig. 3e). D1R antagonism with SCH23390 in the dorsal striatum did not alter performance either in the DMS or in the DLS (Fig. 3). In all cases, the effects were similar for trials to criterion.

Both SCH23390 and raclopride infusions increased latencies to collect the reward across all phases when infused into the DMS (Dose: SCH23390, *F*_1, 113.493_ = 33.828, *p* < 0.001; Raclopride, *F*_2, 192.771_ = 14.706, *p* < 0.001), but not the DLS (ns). Further analysis showed that raclopride caused this effect at both the low and high doses (*p* = 0.002; *p* < 0.001, respectively). Omissions or latencies to respond to the stimuli were not affected after manipulation in any region of the dorsal striatum, neither by raclopride nor by SCH23390 infusions (Table 2).

## Discussion

This study demonstrates dissociable effects on visual serial reversal learning of D2R and D1R antagonists locally infused into the striatum, and shows that the effects of each drug differ fundamentally based on the striatal sub-region targeted and the different learning phases of the task (i.e. the early, perseverative phase versus new learning phases). An important overall finding was that whereas DA receptor antagonism improved reversal-learning performance in the ventral striatum, learning was impaired after drug infusions into the dorsal striatum, clearly showing the different roles of DA signaling within these structures when stimulus-reward contingencies change. This finding is in general consistent with previous data on humans with PD [32, 33] indicating that excess DA activity may often be detrimental for reversal performance in the NAc, whereas intact DA function in the dorsal striatum is necessary for efficient reversal learning, as supported by data from non-human primates [11, 34].

The effects of DA receptor blockade were highly dependent on the phases of reversal learning, as defined by binomial distribution probabilities (cf. [31]) to indicate whether the rats were still being guided by the previous and obsolete stimulus-reward contingencies (significant bias to the previously correct stimulus; early phase; perseveration), at random performance (no bias; mid phase), or had learned to respond in accordance with the new contingencies (significant bias towards the new correct stimulus; late phase). These phases were previously linked to defined brain circuits; e.g., inactivation of the lateral orbitofrontal cortex (OFC) produces increased perseveration in the early phase of visual reversal learning in both marmoset monkeys [35] and rats [36, 37], whereas inactivation of the medial OFC decreases perseveration in visual reversal learning without affecting the later phases of reversal ([37]; but see [38]). In contrast, disrupted function in the medial prefrontal cortex of mice improves the later phases of reversal learning [16], and excitotoxic lesions of the infralimbic cortex impairs late learning in rats [36]. Since the above mentioned prefrontal cortical regions form distinct circuitries and innervate dissociable terminal fields in the striatum [39], it is not unexpected that striatal sub-regions also mediate specific phases of visual reversal learning, both in the present work and from previous reports [12, 40].

The improvements in reversal learning after NAc infusions depended on both the accumbal sub-region and the sub-type of DA receptor, and they were selective for the early phase of reversal learning. Whereas D1R antagonism in the NAcS decreased perseverative errors, this effect was only observed after D2R antagonism in the NAcC. Such a double dissociation refines previous reports showing e.g. that elevated dopaminergic states in the NAc are detrimental for reversal learning [18], and that D2R agonism in the NAc impairs behavioral flexibility [17, 41]. This could be relevant for the DA overdose hypothesis of iatrogenic cognitive impairments associated with dopaminergic drug treatment in PD [42], as our data suggest that such effects are driven by D1R in the NAcS and D2R in the NAcC. However, since the antagonists given here only block endogenous ligands (i.e. DA), our data also suggest that DA signalling at D1R in the NAcS and D2R in the NAcC contribute to perseverative responding in visual reversal learning, perhaps by inappropriately maintaining the previous stimulus-reward association [43] or Pavlovian conditioned approach [44]. Inactivation of the NAcS can also improve various forms of behavioral flexibility, including latent inhibition [45], attentional set-shifting [26] and spatial reversal learning [22, 23, 46]; our results suggest that such effects could be mediated by D1R-expressing neurons.

Additionally, blocking D1R in the NAcC disrupted performance overall by increasing omissions. This effect is similar to what was previously reported after NAcC infusions of higher doses of both raclopride and SCH23390 in rats trained on a visual reversal task [47]. However, it is noteworthy that rats treated with intra-NAcC SCH23390 in our task consistently initiated trials but then failed to respond to either stimulus; again an effect only noticeable in the early phase. While it is possible that D1R antagonism interferes with the processing of visual cues, an alternative interpretation is therefore that such receptor blockade selectively impairs learning from positive feedback by blunting the impact of positive prediction errors, as theorised by Frank and colleagues [48]. Hence, rats in our task could rapidly learn (from negative feedback) that the previously positive stimulus is now incorrect, but, due to the NAcC D1R blockade, not be able to update the value they associate with the previously incorrect, now rewarded stimulus. We recently found some evidence for such an effect of systemic D1R antagonism in visual reversal learning [49].

In the dorsal striatum, D2R antagonism was active in the DMS where it delayed the re-learning of the new stimulus-reward contingencies (mid phase), but did not affect either early or late phases; in the DLS, D2R antagonism impaired reversal learning overall, including the initial (perseverative) phase and during subsequent learning. D1R antagonism showed a lack of effect in both the DMS and the DLS at doses and infusion parameters routinely used in the literature [50]. Hence, D2R antagonism in the DMS and DLS had almost complementary effects with regard to the phase of reversal that was affected. It is plausible theoretically to reconcile this dissociation with evidence that the DMS and DLS mediate different aspects of instrumental learning in both rodents and humans [15]. Whereas the DMS is generally associated with goal-directed behavior, the DLS is thought to mediate habitual, stimulus-response behavior [13]. In this context, it is noteworthy that well-trained visual discrimination may exhibit rule-like or habitual tendencies [51], which need surmounting for reversal learning to proceed. Such top-down executive control over habitual tendencies may implicate cortico-striatal projections. The present data suggest that striatal D2R might play an important modulatory role in controlling habits. These findings for the rat DLS are consistent with recent evidence that the putamen in primates also plays a key role in reversal learning [10, 11]. By contrast, the DMS is implicated in DA-dependent goal-directed behavior and so the modulation of the mid phase, characterised by new learning, by intra-DMS raclopride was predictable. Our data on dorsal-striatal D2R and reversal learning is in accordance with the positive relationship between behavioral flexibility and D2R availability in both caudate and putamen, but not ventral striatum, of vervet monkeys trained in a visual reversal task [11]. This could be relevant also for human conditions such as OCD and substance-use disorder, where reduced D2R binding has been reported [52, 53]. For example, the mixed full/partial D2R agonist pramipexole ameliorated deficits in reversal performance in chronic stimulant abusers with a concomitant normalisation of on-task activation of the caudate nucleus [4].

These findings add to considerable data implicating DA receptors in reversal learning across species by showing that D1R and D2R antagonism can both impair and improve reversal according to the region of the striatum and at the stage of learning this occurs. Of particular interest are two recent studies; Horst and colleagues found that a D2R agonist infused into the caudate nucleus improved serial visual reversal learning at intermediate doses in marmoset monkeys [54], whereas Verharen et al. reported that D1R and D2R agonists impaired probabilistic spatial reversal learning in rats, both after systemic treatment and after local infusions into the ventral striatum [41].

### Limitations

A number of limitations should be borne in mind when interpreting the results from this set of experiments. Firstly, all rats first completed the Latin Square-design experiment investigating the impact of raclopride on reversal learning, and then received the SCH23390 infusions in a cross-over experiment. It is possible that the additional training (three reversals minimum), number of prior infusion events (average 12 infusions during the raclopride experiment) or plastic changes in, e.g., membrane presentation of receptors after exposure to a D2R antagonist altered the impact of subsequent SCH23390 infusions. Next, all rats in this study were male, and it is conceivable that future studies will reveal sex differences in the impact of D2R or D1R antagonism on reversal learning. In addition, it must be noted that SCH23390, although frequently used for experiments targeting the D1R, also shows affinity (as an agonist) at the serotonin 5-HT_2C_ receptor [55], which could in theory contribute to the effects observed after NAcC and NAcS infusions. However, previous reports have suggested no impact on reversal learning after 5-HT_2C_ receptor manipulation in the NAcC [56].

Perhaps more importantly, the D2R antagonist drug employed also has strong dopamine D_3_ receptors (D3R) antagonism properties and, so like many studies employing such drugs we are unable clearly to distinguish between D2R and D3R actions. Furthermore, understanding and dissecting the role of DA signalling is challenging due to the expression of D2R both in pre- and post-synaptic striatal neurons, as well as on striatal GABAergic and cholinergic interneurons [57, 58].

In addition, although the present findings imply that visual reversal learning involves sequential processing in ventral striatal and dorsal striatal domains, more direct evidence would come from monitoring the involvement of all of these regions simultaneously during the course of reversal learning [12].

## Conclusions

The current study elucidates the involvement of DA in reversal learning and suggests that striatal regions differentially modulate this form of behavioral flexibility. Using a serial visual reversal learning task in touchscreen operant chambers, we show that infusions of D1R and D2R antagonists in four striatal sub-regions (NAcC, NAcS, DMS, and DLS) differentially affect distinct phases in reversal learning. These results enhance our understanding of the neural circuits underlying visual reversal learning and could be relevant for cognitive inflexibility in DA-related disorders, such as PD [32], OCD [52] or drug addiction [53].

## Funding and disclosure

This research was funded by a Wellcome Trust Senior Investigator award to TWR (104631/Z/14/Z) and an award from Boehringer Ingelheim to JWD. All experiments were conducted at the Behavioral and Clinical Neuroscience Institute, which was jointly funded by the Medical Research Council and the Wellcome Trust. JSB was supported by a PhD scholarship from the La Caixa Foundation, Spain, and a studentship from Boehringer Ingelheim Pharma GmbH, Germany. LF was funded by a Biotechnology and Biological Sciences Research Council Doctoral Training Partnership. JRN is a full-time employee at Boehringer Ingelheim Pharma GmbH, Germany. JWD has received funding from GlaxoSmithKline. TWR is a consultant for, and receives royalties from, Cambridge Cognition; is a consultant for Unilever and Greenfield Bioventures, had recent research grants with Shionogi and Small Pharma and GlaxoSmithKline and receives editorial honoraria from Springer Nature and Elsevier. The rest of the authors declare no conflict of interest.

## Supplementary information

Supplementary Material. Dorsal and ventral striatal dopamine D1 and D2 receptors differentially modulate distinct phases of serial visual reversal learning
