# Supplementary material for: Dorsal and ventral striatal dopamine D1 and D2 receptors differentially modulate distinct phases of serial visual reversal learning
Source: Neuropsychopharmacology. 2020 Jan 15;45(5):736–44. doi: 10.1038/s41386-020-0612-4 (PMC7075980; doi:10.1038/s41386-020-0612-4)
Supplement: Supplementary file 1 — Supplementary Material. Dorsal and ventral striatal dopamine D1 and D2 receptors differentially modulate distinct phases of serial visual reversal learning [file 41386_2020_612_MOESM1_ESM.docx]

*Supplementary material*

**Dorsal and ventral striatal dopamine D1 and D2 receptors differentially modulate distinct phases of serial visual reversal learning**

Júlia Sala-Bayo^1^; Leanne Fiddian^1^; Simon R. O. Nilsson^1^; Mona El-Sayed Hervig^1^; Colin McKenzie^1^; Alexis Mareschi^1^; Maria Boulos^1^; Peter Zhukovsky^1^; Janet Nicholson^2^; Jeffrey W. Dalley^1,3^; Johan Alsiö^1^*; Trevor W. Robbins^1^*^⸶^

1. Department of Psychology and Behavioural and Clinical Neuroscience Institute, University of Cambridge, Cambridge CB2 2EB, UK

2. Boehringer Ingelheim Pharma GmbH & Co. KG, Div. Research Germany, Biberach an der Riß, Germany

3. Department of Psychiatry, University of Cambridge, Cambridge CB2 2QQ, UK

* These authors have contributed equally

^†^ Author for correspondence: Professor Trevor W. Robbins, Department of Psychology, University of Cambridge, Downing St, Cambridge CB2 3EB, UK. Tel. +44(0)1223 333563; Fax. +44(0)1223 333 564. Email. twr2@cam.ac.uk

**Supplementary materials and methods**

Apparatus

Rats were trained in 28 operant chambers (Med Associates, Georgia, VT, USA), each enclosed within a sound-attenuating wooden box fitted with a fan for ventilation. Each chamber measured 29 x31 x 24 cm with Plexiglas ceiling, front door and back panel. On one wall, they were all equipped with a house light (3 W), pellet dispenser, magazine with light and photocell nose poke detector. The opposite wall was replaced with an infrared touchscreen monitor (29 x 23 cm). The floor was made of stainless bars separated 1 cm from each other with a tray underneath. Access was through a hinged sidewall, secured with a latch during testing.

Surgery

Bilateral 22G guide cannulae (PlasticsOne, Sevenoaks, UK) were implanted in the NAcC, NAcS, aDMS, pDMS or aDLS following standard stereotaxic techniques. Rats were anesthetized using isoflurane in oxygen and secured in a stereotaxic frame fitted with atraumatic ear bars. Anesthesia was induced at 5% and maintained at 2.5% isoflurane. Flat skull was achieved by measuring dorsoventral (DV) from lambda and bregma. All anteroposterior (AP) and mediolateral (ML) coordinates were measured from bregma and DV from dura (Paxinos and Watson, 1998). Coordinates for NAcC guides: AP +1.2, DV -1.9 and ML ±1.9; for NAcS: AP +1.6, DV -1.9 and ML ±0.75; for aDMS: AP +1.2, DV -1.9 and ML ± 1.9; for pDMS: AP -0.4, DV -2.4 and ML ± 2.6; and for aDLS: AP +1.2, DV -2.4 and ML ±3.5 (Table 1). Four metal screws and dental cement secured the guide cannulae to the skull. Obdurators were introduced in the guide cannulae and protected with a dust cap. After surgery, animals were given at least 7 days to fully recover and received 0.3 ml/day orally of 1.5 mg/ml meloxicam (Metacam, Boehringer Ingelheim, Germany) during 3 days post-surgery.

Drugs

The dopamine D2R antagonist raclopride (Tocris Bioscience, Bristol, UK) and the D1R antagonist SCH23390 (Sigma-Aldrich, Dorset, UK) were dissolved in physiological saline. Aliquots were frozen at -80 °C in the quantities required for each testing day.

Intracerebral drug microinfusions

To ensure stable serial reversal performance following surgery, rats were tested for a single reversal learning as a baseline. After these sessions, rats received a mock infusion with injectors lowered in the brain but no solution administered. The following day, rats underwent a retention session following an infusion of vehicle solution to habituate the rats to the infusion procedure. On the following day, rats were infused with raclopride or its vehicle. For intra-striatal microinfusions, doses were 0, 0.1 and 1 μg/ul of raclopride, administered in a Latin-square design, and 0 and 1 μg/ul of SCH23390, administered in a cross-over design. Dose order for each rat was randomized and controlled for baseline performance. However, all animals were infused with raclopride first and received SCH23390 infusions only after the raclopride Latin Square was completed (i.e., although the individual doses within each experiment was counter-balanced, we did not counter-balance between raclopride and SCH23390). Drugs were administered daily before each testing session until rats reached criterion. After criterion, a retention session followed without infusion, and the reversal was considered completed. On the day prior to the following reversal, animals underwent a second retention session, typically after 2 days had relapsed from the previous one. During this second retention session, rats received a vehicle infusion prior to the session. On average, each animal received 4 sessions with infusions per reversal cycle (vehicle on retention + 3 days of drug dose or vehicle during reversal), thus around 12 infusions in the raclopride Latin Square, and 8 infusions in the cross-over study for SCH23390.

Injectors from PlasticOne (28G) extended 5 mm below the guide in the NAcC and NAcS (DV – 6.9 in both cases), 2 mm in the aDLS and pDMS (DV -4.4 in both cases) and 2.5 mm in the aDMS (DV -4.4). During the infusion procedure, rats were gently restrained or allowed to freely move on the lap of the experimenter. Infusions were given in a volume of 0.5 μl over 2 min. The injectors were left in place for 1 min both prior to and after the infusion. Rats were returned to their cage for 5 min before the start of the session.

Histology

Following completion of the behavioral procedures, animals were anaesthetized with a lethal dose of pentobarbital (4 ml/kg; Euthatal, Merial Animal Health Ltd., France) and perfused transcardially with 0.01 M phosphate-buffered saline (PBS) followed by 4% paraformaldehyde (PFA). Brains were removed and post-fixed in PFA for 24 h and dehydrated in 30% sucrose in 0.01 M PBS. Brains were sectioned coronally at 60 μm, mounted and stained with Cresyl-violet to verify injector-tip placement within the different striatal sub-regions. Only rats with correct cannulae placement were included in the analysis.

**Supplementary results**

Effects of D2R and D1R antagonism within the dorsomedial striatum sub-regions

Anterior DMS and posterior DMS did not differ in the modulation of reversal learning after local infusions of D2R antagonist raclopride or D1R antagonist SCH23390.

Following infusions of raclopride, on errors to criterion, there was a non-significant Dose × Phase × Region (F_4, 196.002_ = 0.565, p = 0.689), Phase × Region (F_2, 36.834_ = 0.598, p = 0.555) and Dose × Region (F_2, 196.002_ = 0.264, p = 0.769) interactions (Supplementary Fig. 1A). None of the other variables, including total errors to criterion, trials (data not shown), omissions or latencies had a significant effect (Supplementary Table 1A). Similarly, for errors to criterion by phase, infusions of SCH23390 produced a non-significant Dose × Phase × Region (F_2, 91.999_ = 0.777, p = 0.463), Phase × Region (F_2, 28.676_ = 0.111, p = 0.896) or Dose × Region (F_1, 91.999_ < 0.001, p = 0.984) interactions (Supplementary Fig. 1B). As with raclopride, none of the other variables, including total errors to criterion, trials (data not shown), omissions or latencies had a significant effect (Supplementary Table 1B).


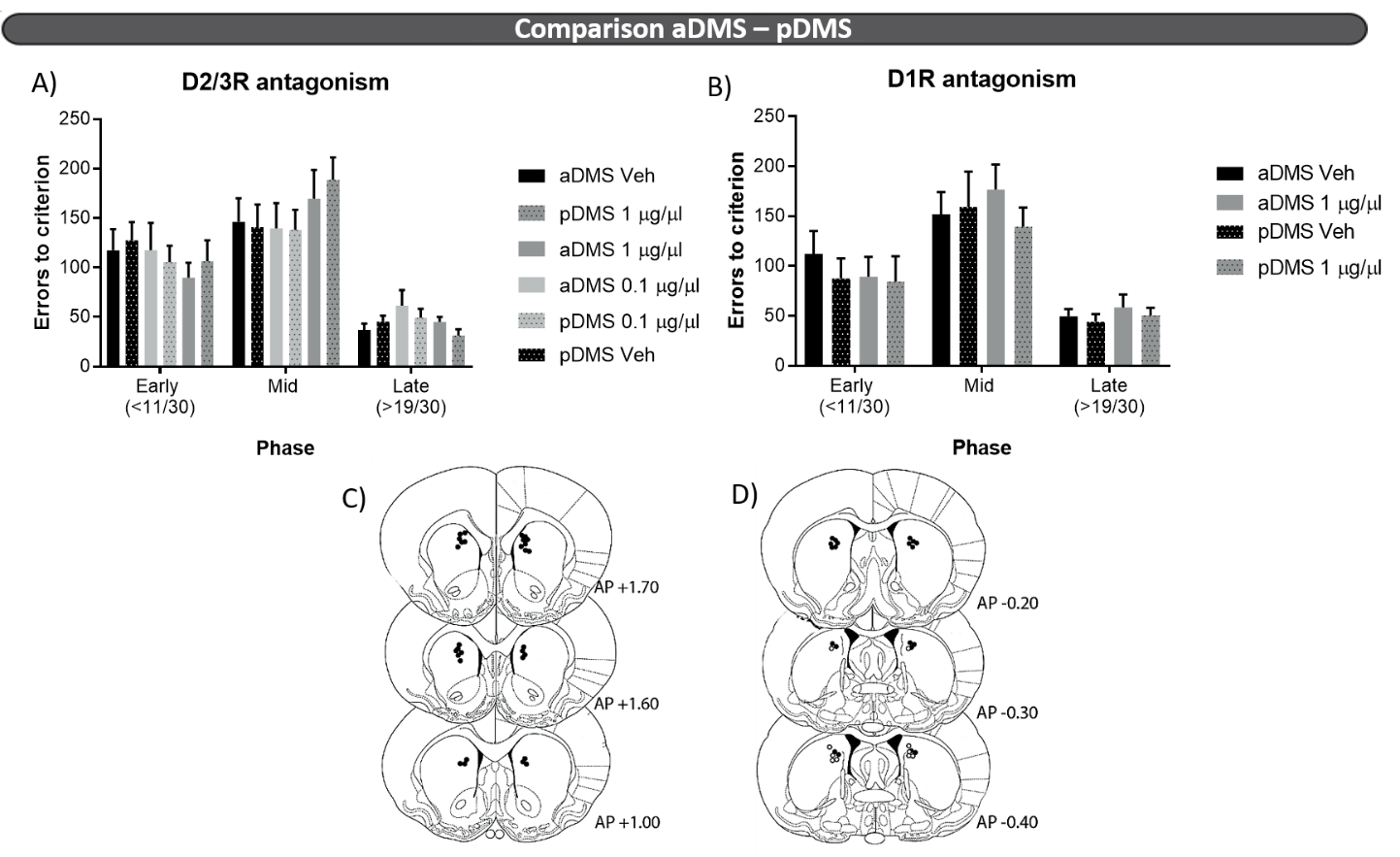

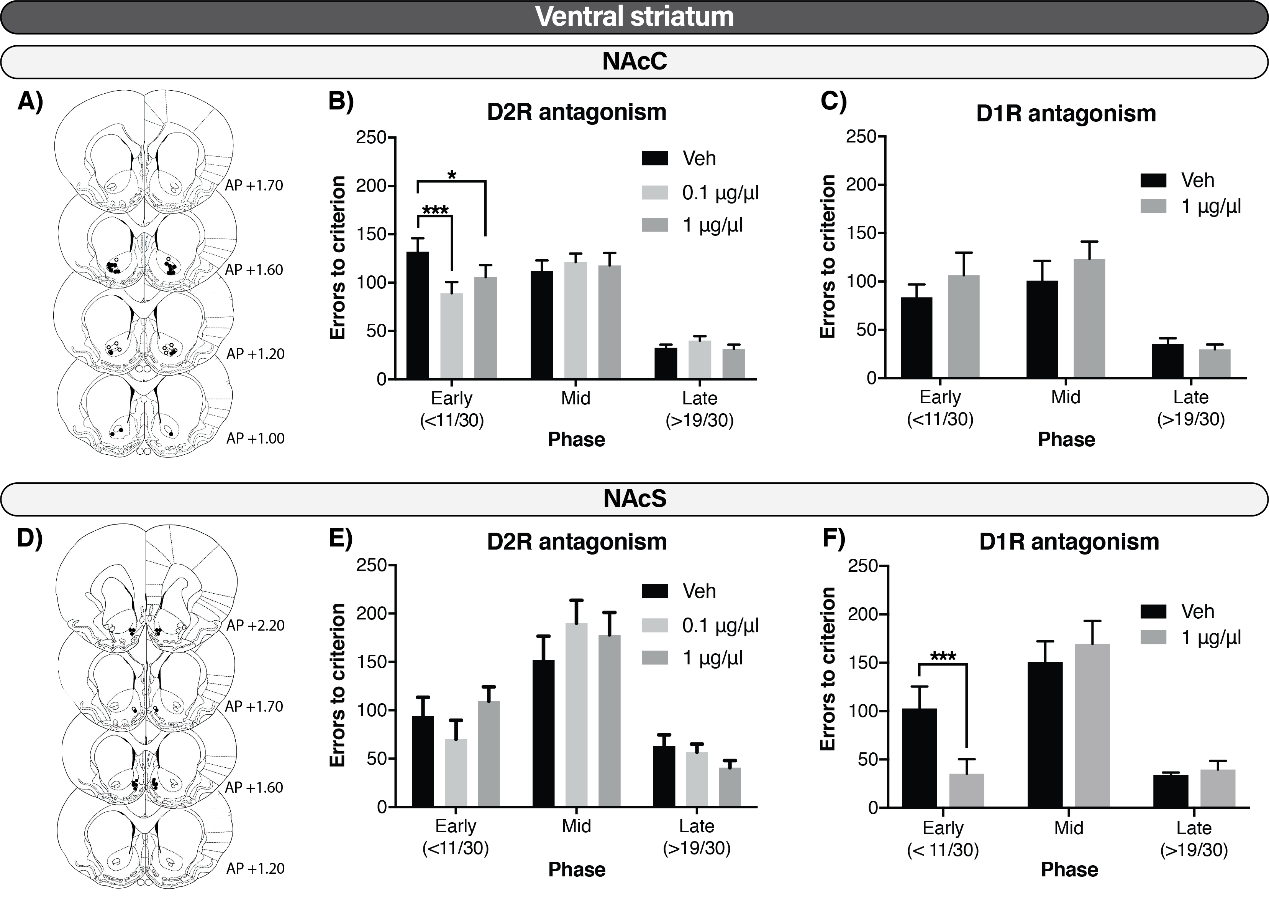


**Supplementary Figure 1.** Comparison of the modulation of aDMS and pDMS in serial reversal learning. Errors to criterion by phase after infusions of A) the D2R antagonist raclopride and B) the D1R antagonist SCH23390. Injector tips placements in the C) aDMS and D) pDMS.

1. **Raclopride**
2. **SCH23390**

| **Region** | **Dose** | **Omissions** | | | **Latency to collect** | | | **Latency to respond** | | |
| --- | --- | --- | --- | --- | --- | --- | --- | --- | --- | --- |
|  |  | **Early** | **Mid** | **Late** | **Early** | **Mid** | **Late** | **Early** | **Mid** | **Late** |
| **aDMS** | **Vehicle** | 1.88 ± 0.81 | 2.06 ± 0.76 | 0.31 ± 0.12 | 3.14 ± 0.05 | 3.05 ± 0.03 | 2.95 ± 0.04 | 3.05 ± 0.03 | 3.04 ± 0.03 | 3.03 ± 0.03 |
|  | **1** | 1.63 ± 0.81 | 2.81 ± 1.12 | 0.88 ± 0.51 | 3.28 ± 0.06 | 3.23 ± 0.06 | 3.19 ± 0.05 | 3.05 ± 0.03 | 3.06 ± 0.03 | 3.09 ± 0.03 |
| **pDMS** | **Vehicle** | 0.00 ± 0.00 | 0.70 ± 0.50 | 0.10 ± 0.10 | 3.17 ± 0.04 | 3.12 ± 0.06 | 3.04 ± 0.06 | 2.96 ± 0.02 | 2.98 ± 0.03 | 2.98 ± 0.03 |
|  | **1** | 0.80 ± 0.47 | 0.50 ± 0.31 | 0.10 ± 0.10 | 3.24 ± 0.09 | 3.22 ± 0.07 | 3.11 ± 0.05 | 2.96 ± 0.02 | 2.97 ± 0.01 | 2.99 ± 0.03 |

| **Region** | **Dose** | **Omissions** | | | **Latency to collect** | | | **Latency to respond** | | |
| --- | --- | --- | --- | --- | --- | --- | --- | --- | --- | --- |
|  |  | **Early** | **Mid** | **Late** | **Early** | **Mid** | **Late** | **Early** | **Mid** | **Late** |
| **aDMS** | **Vehicle** | 1.54 ± 0.52 | 2.13 ± 0.65 | 0.50 ± 0.21 | 3.12 ± 0.05 | 3.07 ± 0.04 | 2.96 ± 0.05 | 3.03 ± 0.03 | 3.03 ± 0.02 | 3.04 ± 0.02 |
|  | **0.1** | 1.29 ± 0.63 | 1.17 ± 0.58 | 0.63 ± 0.19 | 3.16 ± 0.05 | 3.09 ± 0.04 | 2.99 ± 0.04 | 3.04 ± 0.03 | 3.04 ± 0.02 | 3.05 ± 0.02 |
|  | **1** | 3.79 ± 1.97 | 4.17 ± 1.14 | 1.75 ± 0.99 | 3.25 ± 0.05 | 3.15 ± 0.04 | 3.03 ± 0.04 | 3.08 ± 0.03 | 3.08 ± 0.02 | 3.07 ± 0.02 |
| **pDMS** | **Vehicle** | 1.19 ± 0.43 | 0.81 ± 0.23 | 0.13 ± 0.13 | 3.01 ± 0.03 | 3.04 ± 0.04 | 2.92 ± 0.05 | 3.00 ± 0.02 | 2.99 ± 0.02 | 2.97 ± 0.02 |
|  | **0.1** | 0.81 ± 0.43 | 1.13 ± 0.23 | 0.19 ± 0.13 | 3.11 ± 0.30 | 3.10 ± 0.04 | 2.95 ± 0.06 | 2.99 ± 0.02 | 2.97 ± 0.02 | 2.97 ± 0.02 |
|  | **1** | 3.31 ± 1.40 | 1.69 ± 0.69 | 0.50 ± 0.32 | 3.19 ± 0.07 | 3.10 ± 0.04 | 3.07 ± 0.06 | 3.07 ± 0.03 | 3.02 ± 0.03 | 3.05 ± 0.04 |

**Supplementary Table 1.** Effects of microinfusions of the A) D2R antagonist, raclopride (0, 0.1, 1 µg/µl) or B) D1R antagonist, SCH23390 (0, 1 µg/µl), in the aDMS and pDMS in the different phases of visual reversal learning (early, mid and late) as omissions, latency to collect the reward and latency to respond. Latencies are presented as log-transformed. Data are mean ± SEM.
